# Biomedical Journals and Global Poverty: Is HINARI a Step Backwards?

**DOI:** 10.1371/journal.pmed.0040220

**Published:** 2007-06-26

**Authors:** Javier Villafuerte-Gálvez, Walter H Curioso, Oscar Gayoso

## Abstract

N/A

Much has been written about how open access to biomedical journals is vital for researchers in developing countries [1], but so much more needs to be done.

Our experience in Peru with the Health InterNetwork Access to Research Initiative (HINARI), an initiative managed by the World Health Organization that helps promote access to scientific information by providing free (or low cost) online access to major science journals, is not as accessible as hoped for and, in fact, is getting worse. When HINARI launched in 2003, it provided access to more than 2,300 major journals in biomedical and related social sciences [2].

In April 2007, we conducted a review of the first 150 science journals available through HINARI with the highest impact factors on the Science Citation Index [3]. We excluded open-access journals and journals that make online access free to low-income countries (e.g., *The New England Journal of Medicine*, British Medical Journal Publishing Group). We could not access any of the top five journals from major publishers such as Nature and Elsevier-Science Direct. In other words, from the Nature Publishing Group we had no access to *Nature Reviews Cancer*, *Nature Reviews Immunology*, *Nature Reviews Molecular Cell Biology*, *Nature*, or *Nature Medicine*, and from Elsevier ScienceDirect we had no access to *Cell*, *Cancer Cell*, *Current Opinion in Cell Biology*, *Immunity*, or *Molecular Cell*. In addition, we could not access any of the first-level journals from Blackwell, Oxford Press University, Lippincott Williams and Wilkins, or Wiley and Sons. In 2003, all these journals were available.

Our findings support comments received from users over the last 8–10 months at the main library at Universidad Peruana Cayetano Heredia (Oscar Gayoso, personal communication). Students and faculty could not get access to biomedical journals from Nature, Elsevier-Science Direct, Blackwell, Oxford Press University, Springer Science, Lippincott Williams and Wilkins, or Wiley and Sons through HINARI. The collections of journals from the above-mentioned publishers together represent approximately 57% (2,118 of 3,741) of journals that were supposed to be accessible through HINARI, while the remaining 43% accessible were largely composed of open-access journals or journals that make online access free to low-income countries.

Moreover, we have found a significant decrease in the number of users accessing HINARI at our institution. For example, the number of HINARI users has decreased from 12,144 in April 2005 to 5,655 in April 2007, which may reflect the loss of impact of the HINARI initiative at our institution. In contrast, the number of users accessing other databases such as ProQuest and EBSCO has increased over the last few months.

Our findings suggest that we not only have access to a reduced number of biomedical journals on HINARI, but we also have no access to the biomedical journals that have the highest impact factors. The HINARI Web site states that it is still incorporating new journal collections. However, we are afraid that any addition that will not provide access to major publishers (such as the Nature Publishing Group, Elsevier ScienceDirect, or Lippincott Williams and Wilkins) could lack real impact according to HINARI's goals.

Since 2003, Peruvian medical students and health professionals have substantially benefited from access to high-quality scientific information through HINARI. Few medical students and very few researchers in the developing world can pay the usual fee of US$20–US$45 to download one article. Not even some private universities in Peru can afford the minimum journal subscription rates, even though these subscriptions would help the universities to become less isolated from global medical research. Having to pay US$1,000 per year to HINARI has left many public universities in the provinces of Peru without access because they cannot afford it. Even for the Peruvian institutions that are currently paying US$1,000 per year to HINARI, what is the real benefit of their HINARI subscription now?

We fear that the loss of access to many key journals that are published by the major companies could be a major setback to the education of medical students in Peru and perhaps around the world. Furthermore, it could make biomedical research in developing countries like Peru, a key element in fighting poverty, even scarcer.

In conclusion, students and researchers in developing countries such as Peru, working at the frontlines of global health problems, need to access more biomedical journals in order to practice evidence-based health care and conduct high-quality research. The recent loss of access to many key biomedical journals in Peru could be a step backwards. We hope the situation described in this letter might help lend support to the proposal of Godlee et al., who suggested that the World Health Organization and its partners should take the lead in establishing an international collaborative group along the lines of the Global Fund to fight AIDS, Tuberculosis and Malaria to achieve the goal of “Universal access to essential health-care information by 2015” or “Health information for all” [4].
